# The Endoplasmic Reticulum Stress/Unfolded Protein Response and Their Contributions to Parkinson’s Disease Physiopathology

**DOI:** 10.3390/cells9112495

**Published:** 2020-11-17

**Authors:** Cristine Alves da Costa, Wejdane El Manaa, Eric Duplan, Frédéric Checler

**Affiliations:** INSERM, CNRS, IPMC, Team Labeled “Laboratory of Excellence (LABEX) Distalz”, Université Côte d’Azur, 660 Route des Lucioles, Sophia-Antipolis, 06560 Valbonne, France; elmanaa@ipmc.cnrs.fr (W.E.M.); duplan@ipmc.cnrs.fr (E.D.); checler@ipmc.cnrs.fr (F.C.)

**Keywords:** Parkinson’s disease, unfolded protein response, reticulum endoplasmic, genetics

## Abstract

Parkinson’s disease (PD) is a multifactorial age-related movement disorder in which defects of both mitochondria and the endoplasmic reticulum (ER) have been reported. The unfolded protein response (UPR) has emerged as a key cellular dysfunction associated with the etiology of the disease. The UPR involves a coordinated response initiated in the endoplasmic reticulum that grants the correct folding of proteins. This review gives insights on the ER and its functioning; the UPR signaling cascades; and the link between ER stress, UPR activation, and physiopathology of PD. Thus, *post-mortem* studies and data obtained by either *in vitro* and *in vivo* pharmacological approaches or by genetic modulation of PD causative genes are described. Further, we discuss the relevance and impact of the UPR to sporadic and genetic PD pathology.

Parkinson’s disease (PD) is the second most frequent neurodegenerative disorder after Alzheimer’s disease. It is characterized at a histopathological level by the presence of intracellular lesions named Lewy bodies or Lewy neurites according to their shape and by exacerbated cell death of dopaminergic neurons. The deficit in dopamine caused by the *substantia nigra* neuronal loss is translated clinically by uncontrollable tremor, hypokinesia, spasticity, and gait abnormalities. Multiple pieces of evidence indicate that two cellular organelles are strongly linked to the physiopathology of PD: the mitochondria and the endoplasmic reticulum (ER). This review will discuss the role of the ER and the signaling cascades activated by this organelle during ER stress and how this dysfunction could account for the etiology of PD.

## 1. The Endoplasmic Reticulum

The endoplasmic reticulum is a cellular organelle that controls the synthesis, the folding, and the post-transductional modifications of almost one-third of proteins. It is the first compartment of the secretion pathway (ER–Golgi–lysosome) in eukaryotic cells. The ER forms a network of elongated tubules and flattened discs covering a large part of the cytoplasm that extends to the nuclear envelope [1,2]. Considering the key role of this organelle in the development of the unfolded protein response (UPR), we provide a short description of its structure and functions below.

### 1.1. Structure of the ER

The ER is usually categorized as smooth (SER) or rough (RER), depending on its morphology, while its intramembranous space is named lumen. The rough phenotype of the RER is linked to the presence of attached ribosomes at its surface facing cytoplasm. The SER is involved in synthesis of carbohydrates and lipids and the RER in production of membranes and secretory proteins [1,2]. Usually, the SER gathers a tubular network and the RER a series of flattened sacs. More recently, a new classification, which takes into account the structure of the membrane rather than its morphology, has been proposed. According to this new classification, the ER is composed of the nuclear envelope, flattened membrane-enclosed sacs known as cisternae, and an interconnected tubular network [3]. These components of the ER are distinguished by the membrane curve. Thus, the tubules of the ER harbor a more important membrane curvature than that of the leaflets of the nuclear envelope and cisternae. The volume of the ER is cell type-dependent. Nevertheless, the ER occupies a consequent cell volume, allowing it to establish contact sites with several intracellular organelles.

Accordingly, the ER interacts with the mitochondria, the plasma membrane, endosomes, and the endolysosomal system. Thus, the ER associates with the mitochondria via the MAM (mitochondria-associated membrane), allowing the exchange of calcium and lipids between these two key cellular organelles [4]. The ER is also in contact with the plasma membrane via ORA1 (olfactory receptor class A-1 like protein 1), CRACM1 (calcium release-activated calcium channel protein 1) and STIM1 (stromal interaction molecule 1), which are both regulated by calcium and are localized in the plasma membrane and in the ER, respectively [5]. The ER interacts with the endosomes [6] via the sterol binding proteins STARD3 (StAR-related lipid transfer protein 3) and its ER-binding partner STARD3NL (STARD3 N-terminal like protein) [7], allowing the delivery of cholesterol to endosomes. Finally, the ER can also interact with the endolysosomal system via MDM1/SNX13 (mitochondrial distribution and morphology 1/Sorting NeXin 13) [8], suggesting an implication of the ER in autophagy control.

Alone or in coordination with other cell organelles, the ER develops several essential functions that control cellular homeostasis.

### 1.2. ER Functions

The ER contributes to several physiological functions. Notably, it is involved in the synthesis and storage of lipids; the synthesis, folding, and export of proteins; calcium homeostasis; and the metabolism of glucose [4]. The ER is a dynamic organelle that is sensitive to nutriments and that coordinates energetic fluctuations and the firing of the most adequate metabolic response necessary to maintain the cell homeostasis.

#### 1.2.1. Lipid Synthesis

The ER plays an essential role in membrane synthesis, the synthesis of lipid vesicles, and the accumulation of fat for energy storage. Lipid synthesis takes place at the membrane level, at membrane interfaces, and at ER contact sites with other organelles. The lipid precursors synthesized in the ER membrane are then converted into structural lipids, sterols, steroid hormones, biliary acids, dolichols, prenyl donors, and a myriad of isoprenoid species with key functions for cellular metabolism. The ER dynamically modifies its membrane structure to adapt to variations in cellular lipid concentrations. It also grants cholesterol homeostasis [9] and the synthesis of lipid components of the cell membrane, namely, sterols, sphingolipids, and phospholipids [10].

#### 1.2.2. Export of Proteins and Lipids

Most of the proteins and lipids synthetized in the ER must be transported to other cell structures mainly by the secretory pathway. To maintain a constant normal flux, the export of proteins must be strictly regulated and any failure of the process of secretion may, in return, severely impact the structure and function of the ER. The generation of the ER–Golgi COPII (coat complex II) transport vesicles is at the heart of the lipid export process [11], but other mechanisms have also been described. For example, it has been shown that a great quantity of lipoproteins are exported from the ER via another type of vesicle named prechylomicron [12].

#### 1.2.3. Calcium Homeostasis

The ER is the main storage site and it plays a central role in the regulation of Ca^2+^ intracellular levels. Calcium is toxic for most of the metabolic processes, but it is also a key signal mediator of several cellular processes. The cellular calcium levels should be tightly regulated to allow for the proper development of protein folding and a timely specific release of calcium. Certain regions of the ER are implicated in the fine regulation of calcium concentration, notably the contact zones between the ER–mitochondria (MAMs) and ER–plasma membrane. The ER takes advantage of a coordinated cascade of events to control Ca^2+^ concentration at each side of its membrane. First, a calcium pump present in the ER membrane allows for the entry of Ca^2+^ in the ER; next, chaperone proteins bind and buffer the free Ca^2+^; and finally, the ER membrane channels grant the release of Ca^2+^ in the cytosol [13].

#### 1.2.4. Synthesis and Folding of Proteins

The main function of the ER is the synthesis and folding of proteins of the secretory pathway, a process mediated by luminal resident chaperones and foldases. These proteins represent 30% of the proteome and are either addressed to the plasma membrane (ionic channels, transporters, etc.), Golgi apparatus, lysosomes (proteases, lipases, etc.), or secreted (albumin, growth factors, insulin, etc.). Some of these proteins may also stay inside the ER as certain chaperones. The folding process includes a translational and post-translational phase in which a newly synthesized protein in the ribosomes (RER) endures a series of modifications and come across a number of molecular chaperones and foldases that assist its proper folding and issue from the ER. The main modifications taking place during the folding process include the cleavage of the signal peptide by the signal sequence peptidase complex (SPC), N-linked glycosylation, formation of disulfide bonds, pro-isomerization, and oligomerization. All modifications taking place during the folding process may be associated with both translational and post translational phases, except oligomerization, which is a post-translational modification. The detailed steps of the folding process have been reviewed by Braakman et al. [14].

The cell consumes a lot of energy to keep the ionic and electronic environment of the ER perfectly adapted to protein folding. Indeed, the ER grants a much higher calcium concentration and a more oxidizing redox potential than cytosol [15]. The resident ER chaperone proteins are the first elements mobilized by the cellular machinery to catalyze the proper folding of the neo-synthetized proteins and they bind and prevent aggregation during the maturation process. These chaperones include the ER lectins (calnexins and calreticulins) and heat shock proteins (HSPs) of the ER (GRP78/BiP (glucose-related protein 78/binding immunoglobulin protein), HSP70 (heat shock protein 70), and GRP94 (glucose-related protein 94)). GRP78/BiP is the most abundant chaperone of the ER [16,17]. Once partially folded, the proteins are taken over by GRP94, which inserts itself into the heart of the protein via an amphipathic finger. GRP94 is a selective chaperone that allows the correct folding of specific proteins. However, the selectivity criteria of GRP94 are still poorly understood.

Often the folding and structural processing of proteins also involves the co-translational addition of an oligosaccharide. This process called *N*-glycosylation is crucial as it ensures that proteins of the secretory pathway are correctly folded, modified, and assembled into multi-protein complexes in the ER. The *N*-glycosylation also prevents the progression of misfolded proteins into the secretory pathway [18]. When the protein has reached a certain degree of folding, the last glucose must be removed by α-glucosidase II. If the protein has not reached its final folding state (“native fold”), it will be taken over by the glucosyltransferase GGT, re-glycosylated, and again fixed by the chaperones calnexin or calreticulin [19,20]. This cycle can be repeated several times before a protein is properly folded or degraded.

Despite the existence of this sophisticated protein folding machinery, the success rate of protein folding is weak for numerous proteins of the secretion pathway. Proteins not properly folded are not tolerated by the cell and are eliminated by two efficient “control/quality” systems. The ERAD (endoplasmic reticulum-associated degradation) is the main degradation pathway of soluble proteins and reticulophagy, which allows the degradation of non-soluble protein aggregates [21]. The ERAD allows for the comeback of unfolded proteins to the cytosol and their consequent ubiquitylation and degradation by the 26S proteasome [22]. The reticulophagy is a selective type of autophagy that allows for the clearance of the ER by the lysosome [23].

Despite the toughness of the folding capacity of the ER, cells often operate near the limits of their secretory capacity. Thus, a wide range of cellular disturbances can affect the efficiency of protein folding in the ER and lead to an accumulation of misfolded proteins within this organelle. This phenomenon is known as ER stress.

## 2. The ER UPR Response

**Figure 1:**
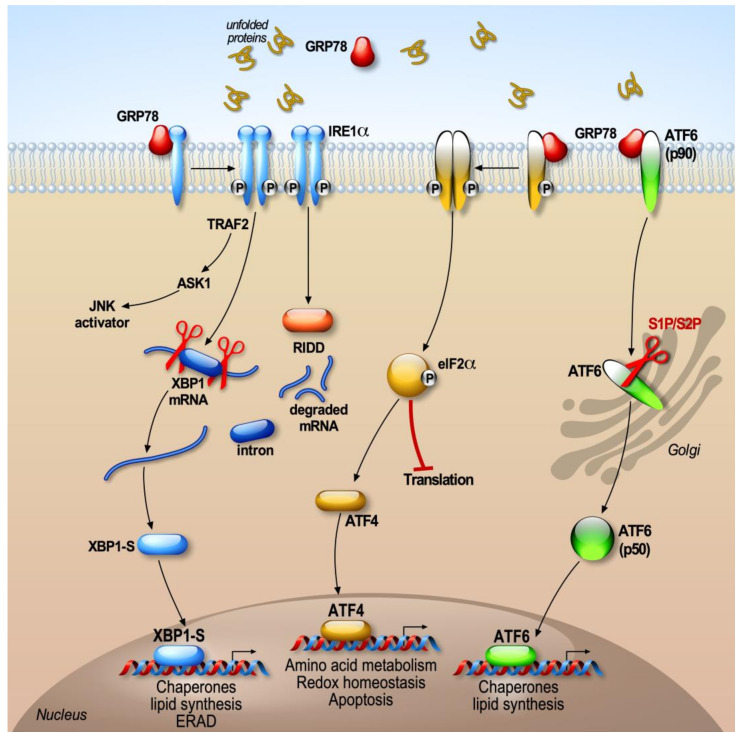
The unfolded protein response (UPR) signaling pathways.

Alterations in ER functions, such as altered calcium levels, increase oxidative stress, or dysfunction of protein N-glycosylation, causing the accumulation of misfolded proteins in the ER, triggering ER stress. In response to the stress of the ER, signaling pathways grouped under the term UPR (unfolded protein response, Figure 1) are activated to circumscribe this stress.

The UPR response concerns an adaptive cellular mechanism that aims to restore the ER homeostasis [24]. The objectives of the activation of the UPR response are (1) to reduce the general synthesis of proteins in order to decrease the accumulation of proteins at the ER lumen, (2) to increase the synthesis of chaperone proteins to facilitate the proper protein folding, and (3) to enhance the translation of proteins implicated in the ERAD machinery in order to foster the elimination of misfolded proteins [25]. The ER stress induces the activation of the three branches of the UPR. The coordinated action of these three branches ultimately boosts the ER folding capacity. Thus, the three branches of the UPR will induce the expression of genes coding either for the chaperones BiP/GRP78, GRP94, and calreticulin, or the enzymes responsible for the establishment of disulfide bridges, in order to improve the ER capacity to properly fold the proteins and thus prevent the accumulation of misfolded proteins [26,27]. In mammals, UPR activation is mediated by signaling pathways involving three sensors located at the ER membrane: IRE1 (inositol-requiring enzyme-1), PERK (protein kinase RNA-like endoplasmic reticulum kinase), and ATF6 (activating transcription factor 6).

The cell activates the IRE1, PERK, and ATF6 pathways, which will differentially modulate downstream effectors capable of binding to specific promoter sequences in order to regulate their target genes. Their luminal domain allows for the integration of the information coming from the lumen of the ER, while their effector cytoplasmic domain mediates the interaction with effectors harboring transcription or translation functions. Thus, the IRE1 effector XBP1 (X box protein 1) and ATF6 bind to sequences ERSE (ER stress response element) [28] in the presence of the cofactor NF-Y (nuclear factor Y), while the transcription factor ATF4 recognizes AARE/CARE (amino acid response element/C/EBP (CAAT-enhancer-binding protein)-ATF) sequences [29]. XBP1 can also recognize UPRE (unfolded protein response) sequences [30].

### 2.1. BiP, the Trigger of the UPR

In the absence of stress, the IRE1, PERK, and ATF6 sensors are kept inactive due to their association with the chaperone BiP/GRP78. Indeed, the luminal sequences of the three ER sensors interact with BiP, which constitutively prevents the activation of these regulatory pathways [31,32]. When misfolded proteins accumulate in the ER, the BiP/GRP78 protein associates preferentially with malformed proteins and dissociates from IRE1, PERK, and ATF6, thus leading to their activation [31,33]. Thanks to this system, the cell can quickly determine the state of reticular stress as a function of the amount of free or bound BiP [34].

Once detached from BiP, “free” IRE1 and PERK form homodimers or oligomers and self-phosphorylate to activate their downstream targets [31]. However, dissociation of BiP from ATF6 unmasks a motif of export of ER [33] that facilitates ATF6 translocation towards the Golgi apparatus [35]. This model of competition of BiP during the activation of the UPR response indicates that BiP is an inhibitor of the UPR response. However, other BiP-dependent or -independent models have been proposed [36]. In addition, it has recently been reported that another ER lumen chaperone, HSP47, is able to displace BiP from IRE1 to promote its oligomerization [37].

### 2.2. PERK Signaling

The PERK signaling cascade is the first branch of the UPR mobilized to cope the stress of the ER. It allows for the punctual reduction of protein synthesis. PERK has been identified in the pancreatic islets of rats as a serine/threonine kinase capable of phosphorylating EIF2α (eukaryotic translational initiation factor 2) [26,38]. PERK is an ubiquitously expressed [38] protein that is structurally composed of a luminal and a cytosolic kinase domain [26].

During ER stress, the dissociation of PERK from BiP triggers the oligomerization of PERK [31] and allows its activation by an autophosphorylation process [39]. Activated PERK phosphorylates EIF2α on serine 51 [26]. This phosphorylation inactivates EIF2α and reduces general protein translation. EIF2α is a subunit of the EIF2 heterotrimer that regulates the first step of protein synthesis by promoting the binding of the initiator tRNA (transfer RNA) to the 40S ribosomal subunits [40,41,42]. Therefore, the blockade of protein translation during ER stress alleviates the folding machinery [43].

Under ER stress conditions, the inactivation of EIF2 leads to the activation of ATF4. ATF4 is a ubiquitous transcription factor that harbors numerous short uORFs (upstream open reading frames) in its 5′ UTR region [43]. The decrease of EIF2 levels upon ER stress leads to a shift of the translation initiation site to the main ORF, resulting in a more efficient synthesis of ATF4 [44]. This process allows increased translation of the transcription factor ATF4, the expression of which is low under physiological conditions [44]. Once synthesized, ATF4 is directed to the nucleus and then binds to the CARE (C/EBP (CAAT-enhancer-binding protein)-ATF-responsive element) of several genes (*ASNS* (asparagine synthetase), *CAT1* (catalase-1), *CHOP* (C/EBP homologous protein), *TRBP3* (transactivation domain binding protein 3), etc.) involved in the control of antioxidant response, protein overload in the ER, and activation of macroautophagy [45].

It is important to note that EIF2α is not the only PERK substrate. Thus, PERK phosphorylates NRF2, allowing its translocation to the nucleus and consequent regulation of genes implicated in anti-oxidant response [46]. The activation of NRF2 by PERK allows the cell to keep its redox potential stable during ER stress.

Finally, PERK is also a mediator of the apoptotic response. In response to acute stress, the PERK pathway modulates several pro-apoptotic factors that contribute to cell degeneration and death if ER stress persists. Thus, even if temporary disruption in protein translation due to phosphorylation of EIF2α is beneficial, sustained blockade of PERK is not compatible with survival of cells [47]. Moreover, the hyperactivation of PERK induces transactivation of CHOP which leads to either a decrease/increase of the expression of anti-apoptotic/pro-apoptotic members of the BCL-2 (B-cell lymphoma 2/BIM (BCL-2-like protein 11) families [47]. The modulation of CHOP by PERK also triggers an increase of oxygenated free radicals by increasing the resident oxidases of the ER such as ERO1A (endoplasmic reticulum oxidoreductase 1 alpha) [48]. CHOP also reduces the total amount of cellular gluthatione and inhibits the translation block linked to PERK-mediated EIF2α phosphorylation [49]. This effect is dependent on GADD34, a phosphatase associated with EIF2α dephosphorylation that is transcriptionally regulated by CHOP [50]. Other pro-apoptotic proteins such as PUMA (p53 upregulated modulator of apoptosis) are also activated by CHOP during acute ER stress [51].

### 2.3. ATF6

ATF6 is an ER transmembrane protein that has a DNA-binding bZIP (basic leucine zip) domain [52]. Under stressful conditions, there is dissociation of the BiP–ATF6 complex, and ATF6 translocates into the Golgi apparatus and is sequentially cleaved by S1P and S2P proteases [53] to produce a cytosolic fragment (ATF6f). This fragment interacts with different nuclear partners, allowing it to upregulate the transcription of ER chaperones and ERAD/EDEM (ER Degradation Enhancing Alpha-Mannosidase-Like) genes [30]. ATF6f collaborates with the cofactor NF-Y (nuclear factor Y) and binds to its targets genes via the recognition of a consensus motif (ER stress response element) [54].

In humans, ATF6 is encoded by two genes: *ATF6A* for ATF6α [54], and *ATF6B* for ATF6β [41]. Despite their high homology, ATF6β behaves as a negative regulator of ATF6α [55]. ATF6’s most important targets are BiP, GRP94, and calnexin [28], but ATF6α also interacts with other bZIP proteins, such as CREB (cAMP (Adenosine 3′5′ Cyclic Monophosphate) Response Element Binding Protein), CREB3L3, NF-Y, yin yang 1, and XBP1 [56,57]. ATF6 and XBP1 are known to act synergistically since they can form heterodimers, allowing ATF6α to act as a stimulator of the transcription of members of ERAD harboring UPRE sites recognized by XBP1 [30]. Indeed, EDEMs and ERAD proteins (3-hydroxy-3-methylglutaryl-coenzyme A (HMG-CoA), reductase degradation 1 (HRD1) and Herp (Homocysteine-induced endoplasmic reticulum protein)) are all transactivated by these heterodimers during ER stress [30].

### 2.4. IRE1 (Inositol-Requiring Enzyme 1)

The IRE1 pathway is the most conserved and best studied UPR pathway. IRE1 has two homologs, IRE1α and IRE1β, which share 40% of structural homologies [58,59,60]. IRE1α is expressed in all cells while the expression of IRE1β is restricted to the gastrointestinal system and to the lining of the pulmonary epithelium [61,62]. The mode of activation of IRE1 is similar to that of PERK. However, besides possessing a kinase activity, the cytosolic domain of IRE1 harbors an atypical endoribonuclease (RNAse) activity. Like other players of the UPR, IRE1 is constitutively inactive when associated to BiP; however, in response to the accumulation of misfolded proteins in the ER, it dissociates from BiP. IRE1 dissociation from BiP allows its dimerization and autophosphorylation on several serine residues. The exact role of these phosphorylations remains unknown, but three of them (Ser724, Ser726, Ser729) have been described as essential for the complete activation of the endoribonuclease function of IRE1 [63]. Importantly, the endoribonuclase activity of IRE1 is responsible for the unconventional splicing of XBP1 mRNA [64,65]. Thus, the excision of 26 nucleotides (intron) in the mRNA of XBP1 causes a shift in the reading frame during the translation of the mRNA, which introduces a new carboxyl domain in the protein XBP1. The splicing of XBP1 mRNA by IRE1 is considered atypical, since it takes place in the cytoplasm rather than in the nucleus, and does not require the consensus sequences used by the spliceosome [66,67]. This atypical splicing makes it possible to generate a stable and active XBP1 protein known as XBP1-S (XBP1-spliced). XBP1-S is a transcription factor composed of a nuclear compartment targeting signal sequence, a transcriptional activation domain and bZIP DNA-binding and dimerization domains.

Depending on the tissue context and stimuli, XBP1 can interact with other transcription factors, forming heterodimers [68]. Under ER stress conditions, XBP1 controls the expression of factors modulating folding, secretion, ERAD, protein translocation in the ER, and lipid synthesis [69,70].

Besides the alternative splicing of XBP1 mRNA, the RNase domain of IRE1 also regulates the stability of several mRNAs. However, unlike XBP1, they are not spliced to produce mature but degraded mRNAs. This process is known under the name of RIDD (regulated IRE1-dependent decay) [27], and it consists in degrading mRNAs directly localized at the ER membrane that do not contain a signal peptide and a specific secondary structure [71]. Thus, IRE1 can degrade its own mRNA in order to regulate its own activation [58], but also other mRNAs, in order to decrease protein synthesis. Ultimately, the role of IRE1 RIDD activity is to control the translation of proteins requiring complex spatial folding that can potentially burden the ER. Interestingly, the unspliced form of XBP1 (XBPu) encodes a protein that acts as a transcriptional repressor of XBP1 [72]. The unconventional splicing of XBP1 mRNA is regulated at different levels and is linked to the transient expression of the unspliced form of XBP1 (XBP1u).

Although XBP1u is highly unstable and rapidly degraded by the 26S proteasome during translation, it can block ER membrane hooked ribosomes through a well-conserved hydrophobic domain, and as a consequence allows the splicing of XBP1 mRNA in the cytosol [73,74,75]. Thus, XBP1u sends its own mRNA to the IRE1 splicing site. The selective targeting of XBP1u mRNA to the ER membrane is mediated by a direct interaction of the ER with the Sec61 translocon [76].

The IRE1/XBP1 pathway is mainly a pro-survival signaling pathway. However, the IRE1 pathway can trigger cell death by apoptosis under certain conditions. Indeed, during ER stress, IRE1 recruits the adapter protein TRAF2 (TNF receptor-associated factor 2) to the ER membrane, leading to the activation of ASK1 (apoptosis signal-regulating kinase 1) and its JNK targets (c-Jun NH2 terminal kinase) and p38 MAPK (mitogen-activated protein kinase) [77,78]. Activated JNK can in turn regulate various members of the BCL-2 family, particularly the pro-apoptotic factors BID (BH3 Interacting Domain Death Agonist) and BIM and the anti-apoptotic factors BCL-2, BCL-XL, and MCL-1 (Induced myeloid leukemia cell differentiation protein) [79,80]. Importantly, p38 MAPK phosphorylates and activates CHOP, which increases expression of BIM and DR5 (Death receptor 5), thereby promoting apoptosis [81,82]. Distinct pro-apoptotic proteins such as BAX, BAK, AIP1 (Actin-Interacting Protein 1), and PTP1B (Protein Tyrosine Phosphatase 1 beta) can interact with IRE1 to facilitate its endoribonuclease activity and thus increase the splicing of XBP1 [83,84,85]. As XBP1 is known as a cytoprotective effector, this regulation suggests that in the early stages of UPR, the possible stimulation of pro-apoptotic factors is not always deleterious and can preserve cellular homeostasis [86,87].

The temporal control of the signaling of the UPR pathway is fundamental in determining the fate of a cell under conditions of ER stress. Although the mechanisms explaining the transition from the adaptive UPR response to the apoptotic UPR response are not definitively established, several models have been proposed to explain how information on the intensity and duration of stimuli is integrated by the cell.

Initially, the UPR response was viewed as a pathway for direct and linear transduction of ER stress levels. However, recent findings have indicated that the three major UPR sensors are finely regulated by post-translational modifications and their binding to various cofactors.

## 3. Implication of the ER UPR in PD Pathology

The link between the UPR and PD’s pathology has been supported by numerous data, which are described below.

### 3.1. Post-Mortem Evidence

The very first study showing a modulation of UPR mediators in PD human brains was provided by Hoozemans and colleagues [88]. They showed an increase of phospho-PERK and phospho-EIF2α protein levels in the *substantia pars compacta* of human PD samples when compared to age-matched controls. An upregulation of BiP in cingulate gyrus and parietal cortex was also demonstrated in dementia with Lewy bodies (DLB) and PD patients by both Western blot and immunohistochemical approaches. Moreover, the accumulation of pPERK (phospho-PERK) in PD human brain was confirmed by immunohistochemical approaches [89].

More recently, Baek and colleagues showed that the mRNA levels of BiP are increased in several brain regions including the cingulate gyrus. However, in contrast to previous data, they showed a decrease of BiP proteins levels [90]. A modulation of GRP78/BiP, ATF4, and CHOP protein levels was observed in SNpc (*substantia nigra pars compacta*) in *post-mortem* human brain samples [91,92]. The accumulation of PDIp, a member of the protein disulfide isomerase family, in PD human brain tissue corroborates the correlation of UPR activation in PD physiopathology and constitutes a neuroprotective adaptive response against ER stress [93]. The PDI family of proteins are linked to disulfide bond formation, reduction, or isomerization of nascent proteins [94,95]. They grant the accurate folding of proteins and are activated during the UPR [96]. Importantly this protein is nitrosylated in PD, leading to its loss of function [97]. An increase of the levels of phosphorylated IRE1 in PD patient samples indicated that the IRE1-XBP1 signaling is associated with PD pathology [98]. Finally, it has been shown that the levels of HERP, a stress response protein associated with ER folding, ER load reduction, and ERAD-mediated degradation of proteins was found to be increased in the *substantia nigra* of PD individuals [99].

### 3.2. Pharmacological Approaches In Vitro and In Vivo

The first evidence of a cause–effect link between ER stress and PD was obtained by pharmacological modulation of UPR *in vitro*. Thus, several studies have shown that treatment of different cellular models, notably the dopaminergic SH-SY5Y neuroblastoma cell line, leads to increased ER stress response. Thus, the parkinsonian inducers 6-hydroxydopamine (6OHDA), 1-methyl-4-phenyl-pyridinium (MPP^+^), and rotenone trigger a significant increase in transcripts associated with the unfolded protein response [100,101,102] in various cell models. This transcriptional regulation was corroborated by the post-transcriptional modulation of the key ER stress kinases IRE1α and PERK and their downstream targets [100]. Microarray analysis of MN9D cells treated with 6OHDA and MPP^+^ confirmed the regulation of transcripts linked to the UPR and showed that both drugs induced a huge upregulation of the pro-apoptotic-linked transcription factor CHOP [101]. Calcium alterations, BiP (decrease), and CHOP (increase) protein level modulation were evidenced in SH-SY5Y cells treated with MPP^+^ [103].

Interestingly, MPP^+^ was shown to induce CDK5 (Cyclin-dependent-like kinase 5)-mediated phosphorylation of XBP1s in rat primary cultured neurons. This phosphorylation favored its nuclear shuttle and transcriptional activity, reinforcing the role of the IRE1-XBP1 pathway in the pathogenesis of sporadic PD [104].

Upregulated levels of phosphorylated EIF2α, BiP, and CHOP expression was evidenced in human and rat dopaminergic models submitted to a 6OHDA treatment [105,106,107]. Moreover, like rotenone, paraquat, 6OHDA, another toxin linked to sporadic PD, was shown to induce apoptosis via the activation of the IRE1α branch of UPR in human and mouse dopaminergic cells [108,109].

Importantly, several animal studies corroborate the *in vitro* data described above. Thus, an induction of the pro-apoptotic IRE1α/caspase-12 branch of the UPR has been shown in the rotenone rat model of PD [110,111]. The systemic delivery of MPTP (1-methyl-4-phenyl-1,2,3,6-tetrahydropyridine) to mice triggers an induction of BiP and CHOP protein and mRNA levels [92], while the intracerebral injection of its metabolite MPP^+^ in rabbit brain leads an activation of ATF6 pathway in SNpc [112]. An induction of the proteins levels of GRP78, CHOP and caspase-12 was reproduced in the model of 6OHDA lesion in rats [113].

Interestingly, the injection of the ER inducer tunicamycin *in vivo* into mice brains caused locomotor deficiency, the death of dopaminergic neurons, and activation of the glia [114]. In addition, high levels of oligomerized α-synuclein was observed in the SNpc of animals injected with tunicamycin. These results suggest that administration of tunicamycin into the *substantia nigra* could be a particularly relevant new pharmacological model of PD for examining the impact of ER stress *in vivo*.

### 3.3. PD Gene Products and Their Influence on the UPR

Genes responsible for autosomal dominant forms of PD.

Of utmost importance, a molecular correlation between PD and the UPR came from studies implying several autosomal-dominant (AD) and -recessive (AR) PD causative genes. Most studies regarding the implication of AD–PD causative proteins in UPR regulation are linked to α-synuclein.

α-Synuclein is a protein encoded by the *SNCA* gene that accumulates in Lewy bodies and Lewy neurites. Several point mutations, duplications, and triplication of the gene have been identified, and multiple *in vitro* and *in vivo* studies indicate that its accumulation triggers its aggregation and thereby induces neurotoxicity [115,116]. The accumulation of aggregated α-syn in the brain and notably its soluble oligomeric toxic form is strongly linked to the etiology of PD [117,118].

The overexpression of α-syn, and thus its aggregated toxic forms, correlates with the chronic activation of multiple branches of the UPR and ER stress-mediated apoptosis. Thus, it has been shown that the overexpression of α-syn triggers the activation of UPR in yeast [119], and that its phosphorylation at serine 129, which is associated to its aggregation and toxicity, leads to an important ATF6 regulation in dopaminergic *in vitro* models [120]. Wild-type and mutated α-syn overexpression in SH-SY5Y cells triggers an alteration in calcium metabolism and an activation of IRE1α-XBP1-signaling pathway [121]. The treatment of differentiated SH-SY5Y cells with oligomeric but not monomeric α-syn leads to enhanced XBP1 splicing, indicating a specific activation of the IRE1-XBP1 signaling pathway by α-syn oligomers [122]. Differentiated 3D5 human neuroblastoma-derived cells overexpressing α-syn show increased levels of GRP78/BiP and phospho-EIF2α [123] in basal conditions, and tunicamycin-induced ER stress leads to accumulation of oligomeric α-syn [123], indicating that ER stress may feed α-syn aggregation and toxicity. α-Syn crowding within the ER induces the activation of the PERK-dependent pathway of the UPR *in vitro* and *in vivo*, an activation process mediated by α-synuclein direct interaction with BiP UPR [124]. α-Synuclein affects ATF6 processing directly via protein–protein interactions or indirectly by means of the reduced incorporation to COPII vesicles. Altered ATF6 processing leads to an impairment of ERAD and increased apoptotic response [125].

Mutations of α-syn that trigger α-syn aggregation affect the UPR response. Thus, the A30P α-syn mutation impacts the mRNA levels of genes involved in the UPR *in vitro* and *in vivo* and induces Golgi fragmentation in LUHMES (Lund Human Mesencephalic) cells [126], while the overexpression of A53T α-syn mutation upregulates the levels of BiP and phosho-EIF2α [127].

The modulation of the UPR by α-syn is not restricted to neurons since mutated α-syn was shown to activate the PERK axis in astrocytes [128]. Considering that astrocytes are involved in various brain functions and support neuronal activity, an activation of UPR by α-syn in these cells may lead to deleterious consequences.

This network of evidence does not make it possible to ascertain whether α-syn neurotoxicity is the cause or the consequence of UPR failure and thus which of them is the primary trigger of PD pathogenesis. Nevertheless, a recent work from Colla et al. in A53T α-syn transgenic mice indicates that the accumulation of α-syn toxic species in the ER is responsible for UPR activation [129] and that the detection of ER-associated α-syn oligomers precedes ER stress response [130], thus suggesting that UPR activation is rather the consequence of accumulation of α-syn in PD. The aggregation of α-syn in the ER has been corroborated by approaches implying (fluorescence resonance energy transfer) FRET biosensors [131].

LRRK2 (leucine-rich repeat kinase 2 gene) is a kinase of the ROCO family [132], the mutations on which are associated to autosomal dominant forms of PD and more than 3% of sporadic PD forms [133,134,135]. The biological functions of LRRK2 remain poorly understood, but a few studies suggest that it is linked to UPR. Thus, studies on LRRK2 subcellular distribution in control versus idiopathic PD revealed that LRRK2 is mainly detected in the ER of neurons and that it co-localizes with two ER-specific markers [136]. The analysis of the contribution of a short hairpin RNA (shRNA)-mediated LRRK2 depletion in SH-SY5Y cells leads to a downregulation of BiP in 6OHDA ER stress conditions, indicating that LRRK2 depletion promotes cytoprotection by modulating the UPR [137].

Moreover, recently it has been demonstrated that LRRK2 may affect mitochondrial bioenergetics by modulating ER–mitochondria tethering via the PERK-mediated ubiquitination pathway [138] and that mutated LRRK2-increased ER stress and apoptosis by disabling the sarco/endoplasmic reticulum Ca^2+^-ATPase (SERCA) in astrocytes [139]. LRRK2-mediated SERCA dysfunction leads to Ca^2+^ overload in the mitochondria.

#### 3.3.1. Genes Responsible for Autosomal Recessive forms of PD (AR PD)

Most studies linking AR PD genes to ER function and UPR are centered around parkin (PRKN), PINK1 (PTEN (Phosphatase and tensin homolog) -induced putative kinase 1) and DJ-1.

Parkin is an E3-ligase [140,141] and transcription factor [142] involved in multiple cellular processes that are affected in PD. Parkin protein is encoded by the PRKN gene, the mutations of which are responsible for most of autosomal recessive juvenile PD [140]. One of the first pieces of evidence linking parkin to ER stress came from *in vitro* studies showing that parkin induced neuroprotection against ER stress [141] and that the Pael (parkin-associated endothelin receptor-like) receptor that is involved in ER stress-mediated apoptosis is a parkin substrate [143]. These studies also demonstrate that the potent ER stress inducer, tunicamycin, leads to an upregulation of parkin mRNA and protein levels that correlates to increased neuroprotection [141]. Moreover, parkin overexpression was found to be protective against Pael ER stress-mediated apoptosis [143].

Interestingly, it has been shown that parkin expression may be induced by either ER or mitochondrial stress via its transcriptional regulation by ATF4. An upregulation of parkin levels protects against mitochondrial failure and cell death, suggesting a functional link between parkin, ER stress, and mitochondrial homeostasis [144]. Moreover, it was shown that salubrinal, an ER stress inhibitor, prevents rotenone-induced apoptosis in SH-SY5Y, corroborating the neuroprotective role of the ATF4–parkin pathway in ER stress triggered by PD inducers [145]. Corroborating the link between ER and mitochondria via parkin, researchers showed that the increase of parkin levels facilitated the crosstalk between these organelles and granted the calcium mitochondrial load to assure cell bioenergetics [146].

We have shown that endogenous and overexpressed parkin are induced by ER stress and that parkin impacts the UPR response via a p53-dependent transcriptional control of XBP1 [147]. These data provide a direct evidence of a role of parkin in neuronal control of the UPR. Of note, parkin-mediated control of ER stress is not restricted to neurons since astrocytes depleted in parkin show increased levels of spliced XBP1, ATF6, ATF4, CHOP, and Ccl2 in response to thapsigargin [148]. Interestingly, it has been shown that the induction of parkin levels may vary according to the cell type since an increased expression of parkin was observed in astrocytes and not primary hippocampal neurons submitted to ER stress [149]. The contrasting data between SH-SY5Y cells and hippocampal neurons may suggest a preponderant function of parkin in dopaminergic neurons. Moreover, in corroborating a cell type-specific induction of parkin by ER stress, it was shown that 2-mercaptoethanol and tunicamycin increased the expression of parkin in SH-SY5Y (H) cells, Neuro2a cells, Goto-P3 cells, but not in SH-SY5Y (J) cells and IMR32 cells [150].

Several *in vivo* models corroborate the impact of parkin to UPR control. Thus, parkin mutant flies show an activation of the PERK branch of the UPR through the establishment of mitofusin bridges between defective mitochondria and the ER [151]. Moreover, drosophila models of parkin overexpression show an enhancement of K48-linked polyubiquitin and reduced levels of protein aggregation during aging [152].

A few studies have implicated PINK1 in the UPR response. PINK1 is a mitochondrial serine/threonine kinase that, in conjunction with parkin, is strongly implicated in the control of mitophagy [153,154]. Mutations of PINK1 are associated to both genetic and sporadic PD cases [155,156] and perturbed mitochondrial homeostasis. Further, the overexpression of the deletion mutant of OTC (ornithine transcarbamylase) (ΔOTC), which induces mitochondrial UPR in mammalian cells [157], leads to an increase of PINK1 protein levels, parkin recruitment, and mitophagy firing without dissipation of mitochondrial potential in HeLa cells [158]. These data indicate that mitochondrial UPR leads to the induction of PINK1–parkin-dependent mitophagy followed by reduced misfolded protein load. Interestingly, PINK1 modulation was also shown to regulate mitochondrial UPR. Thus, mutations in both human and fly PINK1 result in higher levels of misfolded components of respiratory complexes and accumulation of HSP60 [159].

PINK1 was shown to prevent ER-induced apoptosis in mice primary cortical neurons [160], and transcriptomic studies performed in PINK1 knockout aged mice indicated a downregulation of ER stress response genes [161]. Finally, *in vivo* studies in *Drosophila* show that PINK1 mutations are associated with PERK modulation [151].

DJ-1 (PARK7) is a multifunctional protein [162] considered as a mitochondrial oxidative stress cellular sensor that interestingly harbors chaperone properties [163]. In addition to its key mitochondrial function, downregulation of DJ-1 was shown to affect ER mitochondria contacts in SH-SY5Y differentiated cells [164]. Corroborating these data, DJ-1 overexpression was shown to overcome the p53-induced mitochondrial calcium uptake failure and the perturbations in ER–mitochondria tethering [165]. Overexpressed and endogenous DJ-1 proteins protect against ER stress induced by thapsigargin and tunicamycin in Neuro 2a cells [166].

DJ-1 regulates and is regulated by UPR signaling pathway members. Thus, DJ-1 regulates the UPR and apoptotic response through the increase of ATF4 signaling in stress conditions [167] and is transcriptionally regulated by XBP1. Thus, we have shown that XBP-1 directly binds to its promoter, leading to its upregulation [147]. Finally, it has been shown that oxidized DJ-1 binds to R-HSP5 and favors the elimination of misfolded cargo proteins by autophagy in oxidative stress conditions [168].

Among genetic PD, PARK20 is a rare autosomal recessive juvenile Parkinson’s form due to mutations in the phosphatidylinositol phosphatase, synaptojanin1 (Synj1) [169,170]. PARK20 fibroblasts show alterations in the exit machinery of the ER and Golgi trafficking. These alterations lead to the activation of the PERK branch of UPR due to the accumulation of cargo proteins in the ER [171].

Finally, mutations in PLA2G6 (calcium-independent phospholipase A2), which are linked to PARK14-linked young-onset dystonia-parkinsonism syndrome with recessive inheritance [172] were shown to upregulate GRP78, IRE1, PERK, and CHOP protein levels *in vivo* [173].

#### 3.3.2. PD Risk Factors

Glucocerebrosidase (GCase, GBA) is a lysosomal enzyme encoded by the GBA gene that is considered an important risk factor to PD [174]. Mutations in GCase are associated to α-syn accumulation due to an impairment of its CMA (chaperone-mediated autophagy)-mediated degradation [175]. *Post-mortem* analysis of brains of Lewy bodies dementia (LDB) patients carrying GBA1 mutations show alterations on protein levels BiP and HERP, indicating abnormal UPR response [176]. Horowitz’s team has shown that mutations of GCase lead to their retention in the ER and subsequent activation of the UPR in the *Drosophila* model [177]. Moreover, they showed that the activation of UPR, illustrated by increased mRNA levels of XBP1s and Hsp-70, may be reversed by ambraxol, a GCase chaperone [178].

Even if it is still debated, high-temperature requirement A2 (HTRA2/Omi/PARK13) is often considered as a PD risk factor [179,180]. HTRA2 is a serine protease with strong homology to the *Escherichia coli* HTRA2, that are important to bacterial survival at high temperatures. Considering that bacterial HTRA2 is involved in the elimination of misfolded aggregated proteins, it is not surprising that HTRA2 is functionally linked to the UPR. Thus, it has been shown that HTRA2 depletion/invalidation in SH-SY5Y and immortalized mouse embryonic fibroblasts (MEFs) triggers a decrease of the pro-apoptotic CHOP protein in 6OHDA stress conditions [181,182]. Interestingly, it has been shown that HTRA2 is induced by tunicamycin *in vitro*, indicating that Omi is activated by ER stress [183].

### 3.4. UPR Gene Products and Their Contribution to PD

Several studies have demonstrated the impact direct of UPR key players to PD physiopathology. Thus, the overexpression of ATF4 by rAAV (Recombinant adeno-associated virus) approaches in a human α-syn rat model of neurodegeneration triggered a severe nigrostriatal cell death due to an activation of caspases 3 and 7 [184].The depletion of XBP1 by shRNA approach in the *substantia nigra* of adult mice triggers chronic stress of the ER and the specific degeneration of dopaminergic neurons. Conversely, rescue of XBP1 level by gene therapy increases neuronal survival and reduces striatal denervation induced by 6OHDA treatment [185]. This study showed the crucial role of the transcription factor XBP1 in controlling the survival of dopaminergic neurons and the vulnerability of dopaminergic neurons to misfolded proteins. Similar results were obtained in mice after administration of MPTP, or in neuroblastoma cell lines treated with MPTP or proteasome inhibitors [186]. In both cases, the overexpression of XBP1 protects the dopaminergic neurons. Interestingly, several studies have shown that these adaptative responses can be stimulated by preconditioning treatments that confer resistance to a subsequent toxic challenge, a phenomenon called “hormesis” [187,188]. Thus, Mollereau and colleagues demonstrated that the preconditioning of the ER leads to neuroprotection in animal models of PD [189,190]. Interestingly, it has been shown that in the XBP1 conditional knockout animal model, XBP1 depletion pre-conditions dopaminergic neurons to stress, rendering them more resistant to 6OHDA treatment. This protection is accompanied by an increase in the expression of markers of the adaptive response of UPR in SNpc. This preconditioning effect is similar to that demonstrated in mice and *Drosophila* by pharmacological approaches where low doses of tunicamycin selectively induce an adaptive UPR response, involving the expression of XBP1-S and not the apoptotic factor CHOP, offering protection of dopaminergic neurons against 6OHDA challenge [191].

Atypical XBP1 splicing is catalyzed by endoribonuclease IRE1 and RTCB1 (RNA 2′,3′-cyclic phosphate and 5′-OH ligase)-ligase [192]. This ligase has been shown to protect dopaminergic neurons from the effects of overexpression of α-syn in *Caenorhabditis elegans*. This observation made it possible to discover a functional relationship between XBP1 and this ligase in the regulation of neuroprotection against proteostatic stress in these neurons [193]. Furthermore, XBP1 has been shown to be not only be protective when delivered to dopaminergic cells by viral transduction but also when transfected into neural stem cells [194]. In these cells, transfection of XBP1 leads to increased survival and improved motor deficits in rat models of PD, injected with rotenone [194]. One of the functions of XBP1 is to associate with ATF6f to enable transcription of the BiP chaperones. Overexpression of this chaperone also protects dopaminergic neurons and improves motor performance in rat models of PD, induced by direct injection of (adeno-associated virus) AAVs encoding the human form of α-syn into SNpc [195]. This protection is accompanied by an overall reduction in the stress response of ER [195]. Age-related decline in BiP or siRNA expression against BiP has also been shown to increase the vulnerability of neurons to α-syn in the same model of PD [196].

3-Hydroxy-3-methylglutaryl-coenzyme A (HMG-CoA) reductase degradation 1 (HRD1), a key player of the ERAD machinery, inhibits cell death induced by 6OHDA in SH-SY5Y cells [197]. Furthermore, CHOP invalidation in mice leads to the protection of dopaminergic neurons against 6OHDA [198], and ATF6 depletion fosters neurodegeneration and ubiquitin accumulation upon chronic injection of mice with MPTP/probenecide [199].

Overall, the studies described above indicate that the genetic modulation of UPR players may lead to novel therapeutic strategies based on the development of pharmacological modulators of gene products of the UPR.

## 4. Concluding Remarks

**Figure 2:**
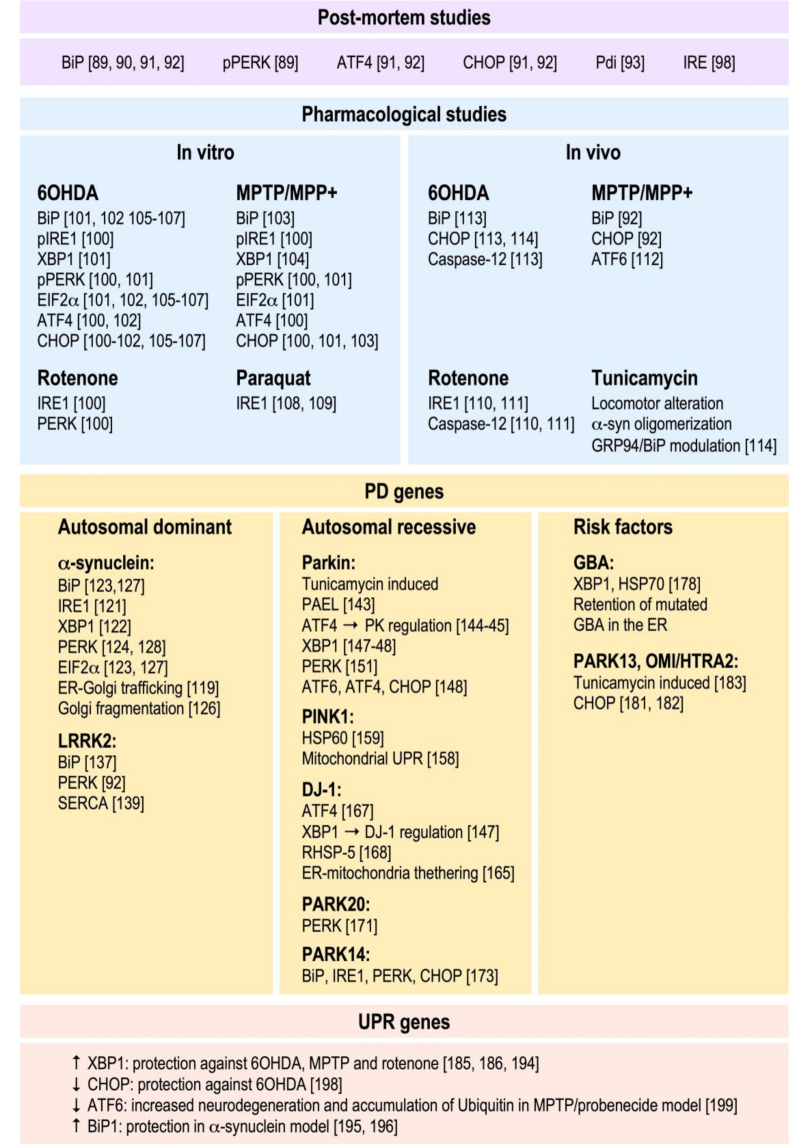
Evidence of the implication of UPR in Parkinson’s disease (PD) physiopathology demonstrated by *post-mortem* analysis and *in vitro* and *in vivo* pharmacological/genetic studies. Reference numbers are provided in brackets.

The numerous studies described above and resumed in Figure 2 highlight the importance of the ER UPR in the physiopathology of PD. They indicate that all branches of the UPR are likely implicated in PD etiology, but the exact chronology of their activation and hierarchy of their pathogenic weights in human brain remain to be established. It is worth noting that the studies implying PD gene modulation in cellular and animal models have strongly contributed to the delineation of UPR signaling cascades underlying neurodegeneration in PD and have reinforced the functional link between the ER and mitochondria. Several studies have highlighted the importance of mitochondrial UPR and the MAMs in this cellular crosstalk. The implication of mitochondrial UPR in PD has been recently reviewed [200,201]. Interestingly, the main pieces of evidence linking mitochondrial UPR to PD pathology came from functional studies linked to PD-related proteins. Notably, it has been shown that misfolded α-synuclein accumulates in the mitochondria [202,203]; that PINK1 interacts with TRAP2, HTRA2, and HSP60 [204,205,206]; and that HTRA2 depletion leads to increased levels of CHOP [182]. It also remains unclear as to whether the UPR dysfunction is rather a cause or consequence of PD; however, there is a general consensus that short and mild UPR activation is beneficial while its sustained activation would be deleterious.

Finally, as a corollary of these fundamental studies that put the UPR at the frontline of cellular dysfunctions taking place in PD, many applied/therapeutic works have recently emerged and are reviewed in [207,208,209]. These works indicate that the development of either pharmacological or genetic strategies to increase the buffering capacity of the proteostasis network may be clinically relevant at short- to mid-term levels. Future fundamental studies should contribute to a better understanding of the UPR mechanism dysfunctions in PD and allow for the development of new therapeutic approaches.
